# Current advances in noninvasive methods for the diagnosis of oral squamous cell carcinoma: a review

**DOI:** 10.1186/s40001-022-00916-4

**Published:** 2023-01-27

**Authors:** Shan Wang, Mao Yang, Ruiying Li, Jie Bai

**Affiliations:** 1 Department of Oral Pathology, School of Stomatology, Hainan Medical College, Haikou, 571199 People’s Republic of China; 2 Department of Stomatology, The Second Affiliated Hospital of Hainan Medical University, Haikou, 570216 People’s Republic of China; 3 West China School of Stomatology, Sichuan University, Chengdu, 610041 People’s Republic of China; 4 Department of Ophthalmology, The Fourth Affiliated Hospital, Zhejiang University School of Medicine, Yiwu, 322000 People’s Republic of China

**Keywords:** Oral squamous cell carcinoma, Diagnosis, Liquid biopsy, Optical detection systems, Oral brush cytology, Microfluidic detection

## Abstract

Oral squamous cell carcinoma (OSCC), one of the most common types of cancers worldwide, is diagnosed mainly through tissue biopsy. However, owing to the tumor heterogeneity and other drawbacks, such as the invasiveness of the biopsy procedure and high cost and limited usefulness of longitudinal surveillance, there has been a focus on adopting more rapid, economical, and noninvasive screening methods. Examples of these include liquid biopsy, optical detection systems, oral brush cytology, microfluidic detection, and artificial intelligence auxiliary diagnosis, which have their own strengths and weaknesses. Extensive research is being performed on various liquid biopsy biomarkers, including novel microbiome components, noncoding RNAs, extracellular vesicles, and circulating tumor DNA. The majority of these elements have demonstrated encouraging clinical outcomes in early OSCC detection. This review summarizes the screening methods for OSCC with a focus on providing new guiding strategies for the diagnosis of the disease.

**Supplementary Information:**

The online version contains supplementary material available at 10.1186/s40001-022-00916-4.

## Introduction

Oral cancer, one of the most common types of malignant neoplasms, poses a significant economic and clinical burden worldwide [1]. Despite advances in its treatment, the 5-year survival rates of afflicted patients have remained constant over the past decades, owing to delayed diagnosis of the disease [2]. According to a World Health Organization report, the mortality rate for oral cancer within 5 years of diagnosis is 45% (for all stages of diagnosis combined) [3]. By contrast, the survival rate is 80–90% if the disease is detected early in its development. Unfortunately, because of insufficient public awareness and screening methods, the early detection of most of these cancers remains difficult to achieve, thereby leading to the poor prognosis and low survival rates [4]. Oral cancer is characterized by its insidious onset, difficult diagnosis, and rapid progression and is often accompanied by metastasis and disabling treatment. The high mortality and morbidity rates associated with the disease highlight the need for an effective screening method and the development of early diagnostic tools [5]. Currently, routine oral examination (visual and tactile inspection of accessible oral structures), together with tissue biopsy, remains the gold standard for diagnosing potentially malignant diseases (PMDs) and oral squamous cell carcinoma (OSCC). However, this method also possesses certain limitations, such as sampling bias, which can result in underdiagnosis or misdiagnosis, especially for multifocal lesions [6]. Therefore, there is an urgent need to explore noninvasive, rapid, and economical screening methods with high enough sensitivity and specificity for the early diagnosis of oral cancer. Herein, we review the current status on research into noninvasive diagnostic methods, such as liquid biopsy, optical detection systems, nano detection technology, microfluidic systems, and artificial intelligence approaches. The strengths and weaknesses of the systems are discussed as well as their potential application for the early diagnosis of OSCC.

## Liquid biopsy

The use of liquid biopsy for cancer screening, as well as patient stratification and monitoring, has been documented extensively [7]. Its use in OSCC is particularly stressed upon, as this is a highly heterogeneous tumor that necessitates molecular characterization for its effective monitoring and management. In addition to blood, other bodily fluids, such as urine, saliva, seminal fluid, pleural effusion, cerebrospinal fluid, sputum, and stool samples, can be used for liquid biopsy [8]. For OSCC, blood and salivary biomarkers are discussed in this review.

**Fig. 1:**
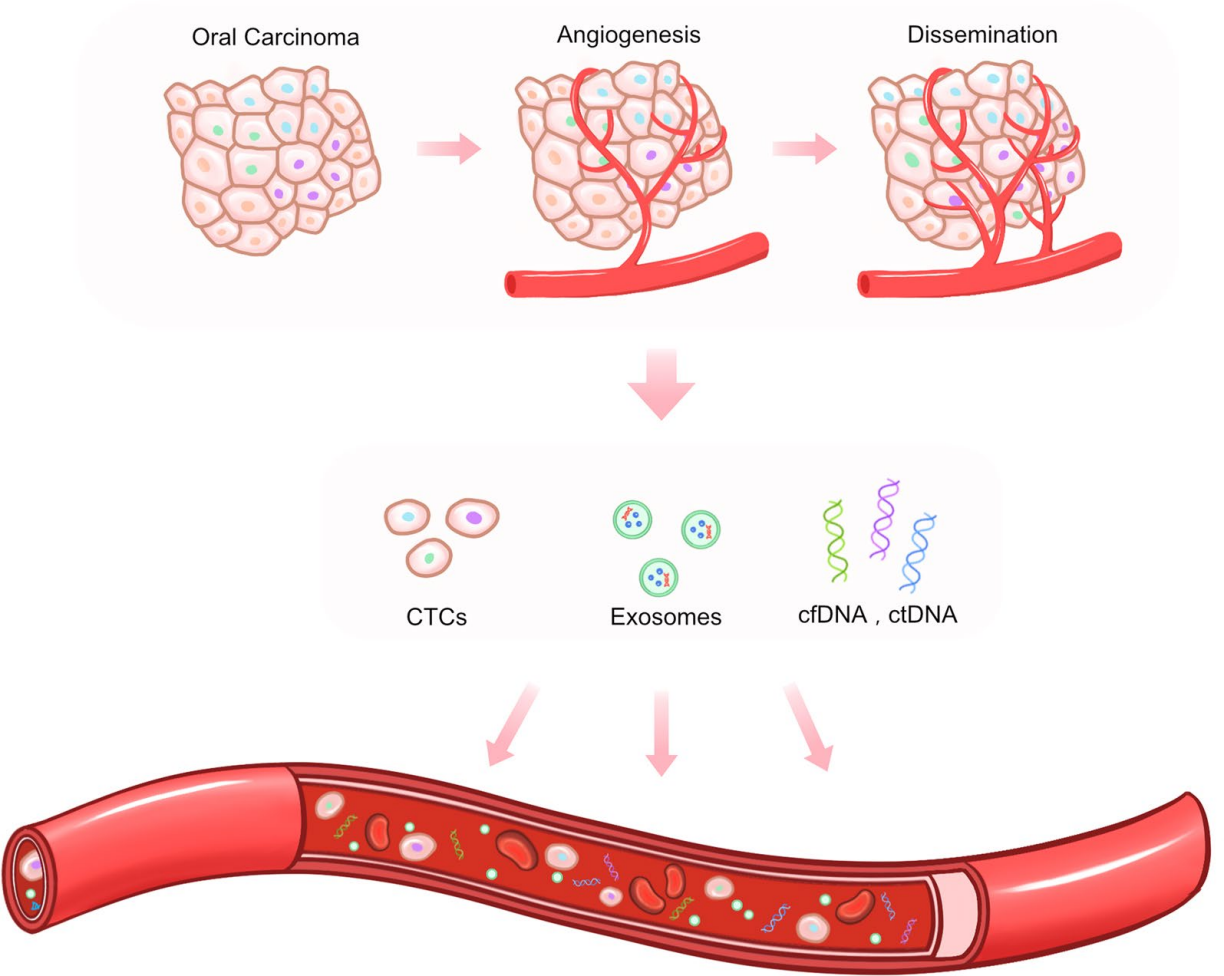
Schematic representation of CTCs, cfDNA, ctDNA, and exosomes for achieving personalized medicine in the diagnosis, prognosis, and treatment monitoring of oral cancer. Individual nuanced and unique characteristics of CTCs, cfDNA, ctDNA, and exosomes can be detected in the carcinogenesis, angiogenesis, and dissemination processes of OSCC

Blood and salivary biomarkers include circulating tumor cells (CTCs), cell-free DNA (cfDNA), circulating tumor DNA (ctDNA), and exosomes, among other molecules [9]. CTCs, which carry an intact viable nucleus and are positive for both cytokeratin and epithelial cell adhesion molecule (EpCAM) and negative for the CD45 molecule, are shed from a primary tumor into the vasculature [10]. These cells are detected in metastatic carcinomas but are extremely rare in nonmetastatic disease [11] (Fig. 1). They not only promote the development of metastasis but can also colonize the site of the primary tumor, supporting its growth via a process known as tumor self-seeding [12, 13]. CellSearch is the most commonly used test system for capturing and enumerating CTCs and is the only platform approved by the US Food and Drug Administration (FDA) for the prognosis of breast, prostate, and colorectal cancers [14]. The CellSearch system consists of an automated instrument for capturing and immunostaining CTCs (AutoPrep; Veridex) and a semiautomated fluorescence microscope for scanning and visualizing the results (CellSpotter Analyzer; Veridex). Samples are processed with the CellSearch Epithelial Cell Kit (Veridex). DAPI- and cytokeratin-positive (but CD45-negative) cells with a diameter of at least 4 um are designated epithelial cells that function as a surrogate for tumor cells [15]. Studies on patients with head and neck squamous cell carcinoma have demonstrated that the individuals without detected CTCs in their blood have a significantly higher probability of disease-free survival [16]. CTCs have encouraging clinical relevance for further investigation compared with markers with very low sensitivity. The use of CTC counts for predicting tumor relapse, especially the risk of early locoregional relapse, may become feasible. The phrase “molecular characterization of CTCs” refers to the identification of potential targets for individualized therapies, as well as the use of repeated CTC assessments in individual patients for treatment surveillance [17].

All cell types frequently produce cfDNA, which is derived from necrotic or apoptotic cells. By contrast, ctDNA is defined as fragmented tumor-derived DNA that is found in the circulatory system and is not associated with cells (i.e., cell free). They are the tumor-derived component of circulating cfDNA, the latter of which is the total DNA shed into the blood and biological fluids during physiological and pathologic apoptosis and necrosis [18]. Lin et al. [19] reported that the plasma cfDNA level was significantly higher in patients with OSCC than in the control group. The plasma cfDNA concentration is associated with an advanced tumor stage, cervical lymph node metastasis, and the tumor size. Higher plasma cfDNA levels have been linked to poor prognosis in OSCC. However, an increased cfDNA level is not a hallmark of cancer, and there is no set cut-off number in any given patient that can be used to quantify the tumor through the ctDNA. This constraint can be circumvented by examining tumor-specific modifications, such methylations and mutations [20].

Exosomes, which are small membrane sacs ranging from 40 to 150 nm in diameter, are a type of lipid bilayer membrane [21]. These extracellular vesicles contain a variety of parent cell-derived bioactive substances, such as proteins, lipids, mRNAs, noncoding RNA, genomic DNA, and cDNA, depending on the mechanism of biogenesis, cell type, and physiological circumstance [22]. Li et al. [23] determined that exosome-mediated paracrine miR-34a-5p promoted the proliferation and metastasis of oral cancer cells. Wang et al. [24] reported that OSCC exosomes promoted angiogenesis in oral cancer by regulating EFNA3-targeting miR-210-3p through the PI3K/AKT pathway. Xiao et al. [25] observed that M1-like tumor-associated macrophages activated by exosome-transferred THBS1 promoted the malignant migration of OSCC. These results indicate that exosomes play an important role in tumorigenesis and cancer cell invasion and metastasis. Recent evidence suggests that tumor exosomes contribute to immunosuppression and promote tumor development and progression. For example, it was found that tumor exosomes could communicate with immune cells within the tumor microenvironment via immunosuppressive (protumor) and immunostimulatory (antitumor) signals [26]. Zlotogorski et al. [27] reported that exosomes isolated from patients with oral cancer exhibited differential expression of various exosome markers (CD63, CD9, and CD81) compared with those isolated from healthy individuals. Therefore, a significantly reduced level of CD9 and CD81 expression, rather than an ambiguous increase in CD63 expression, can be used as an indicator of oral cancer, even in the early stages of the disease. Busso-Lopes mapped the proteomic, microRNA (miRNA), metabolomic, and lipidomic profiles of extracellular vesicles derived from human primary tumor (SCC-9) cells and matched lymph node metastatic (LN1) cells. Their integration of the multi-omics data with results from the analysis of multiple public databases revealed that a low abundance of seven hub proteins (ALDH7A1, CAD, CANT1, GOT1, MTHFD1, PYGB, and SARS) was correlated with a poor prognosis in oral cancer [28].

**Fig. 2:**
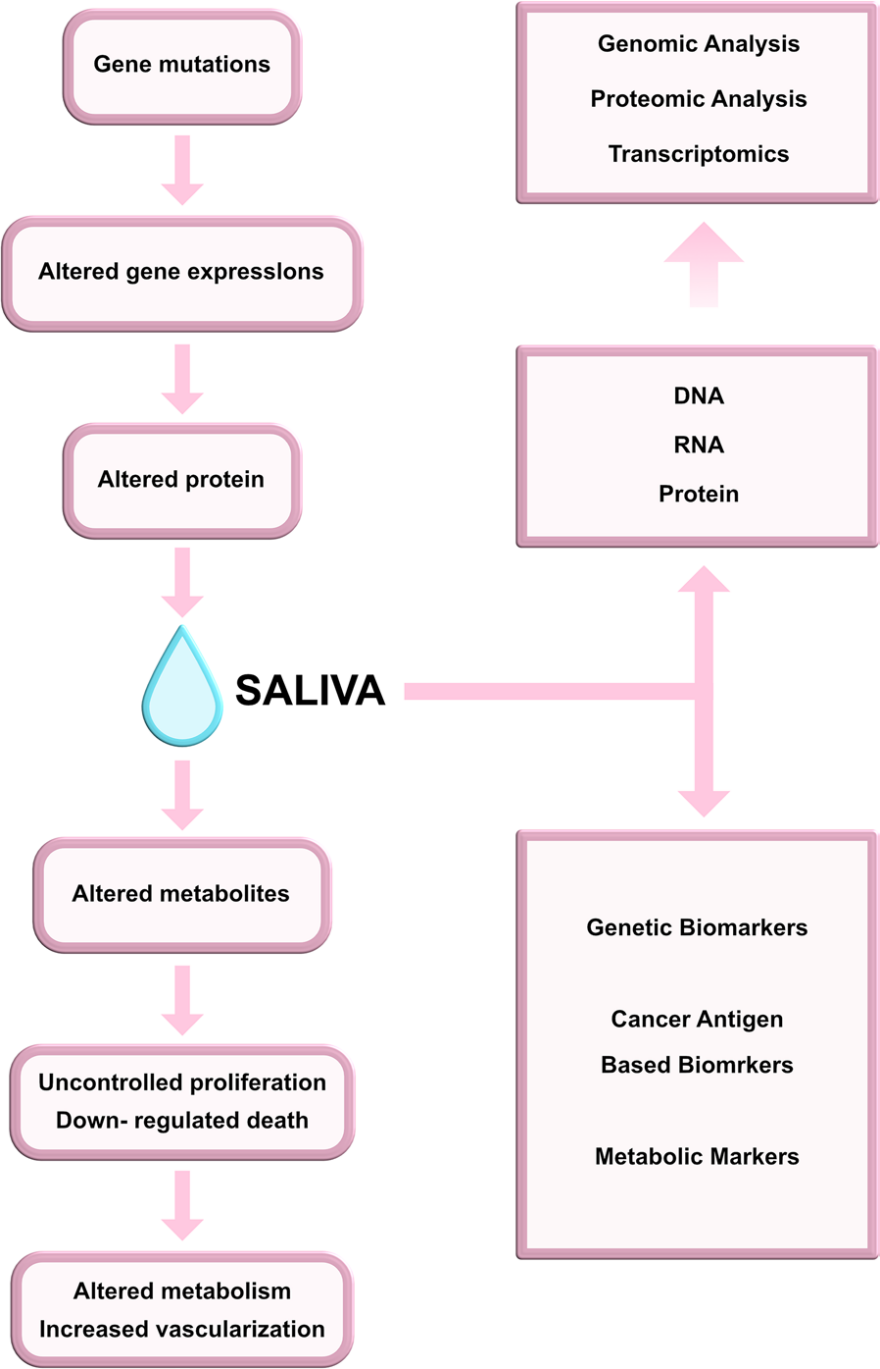
Clinical utility of saliva and the potential of salivary biomarkers in revealing carcinogenesis [29]

Cancer cell transformation and progression are both complicated events that involve the upregulation and downregulation of numerous genes required for cell proliferation, differentiation, and death. As a result, protein analysis is needed to accurately predict marker function [29]. Saliva is an inexhaustible biological fluid that can be used for noninvasive measurements. It contains components, such as DNA, RNA, proteins, metabolites, and microbiota, which can be used for diagnostic purposes, thereby providing an unprecedented wealth of genetic information [30]. Salivary biomarkers can be viewed from several perspectives, such as genomics, proteomics, metabolomics, and microbiomics (Fig. 2) Additionally, focus should be placed on comprehensive analysis of the integrative multi-omics module, where the multiplex network represents such systems effectively and encodes far more information than isolated networks [31, 32].

The OSCC genome contains numerous alterations, such as frequent oncogenic drivers and novel therapeutic candidates. The inclusion of genomic analyses in prospective clinical trials is important, as these findings will be useful if they are extended to a well-annotated and clinically relevant cohort [33]. Guerrero-Preston et al. [34] indicated that hypermethylation of the NID2 and HOXA9 promoters in tissue and salivary cells can be used as biomarkers for the prevention and early detection of OSCC. Lin et al. [35] reported that two of four significantly mutated OSCC-specific genes (*CUL3* and *ZFP36L2*) exerted strong antiproliferative effects on the cancer cells. The other two significantly mutated genes (*PTCH1* and *ATF5*) may play key roles in other processes, such as cell migration, invasion, and epithelial–mesenchymal transformation. Using detailed biochemical, cellular, and animal experiments, those authors further confirmed that both CUL3 and ZFP36L2 are lineage-specific tumor suppressors in OSCC [35].

Cumulative salivary proteome data highlight the potential use of salivary biomarkers as early diagnostic and screening tools for oral tumors. The most abundant proteins in saliva are alpha-amylase, cystatin, proline-rich peptide, serum albumin, and mucin [36]. Depending on the biomarker type, potential salivary biomarkers can be studied using methods, such as the enzyme-linked immunosorbent assay (ELISA), radioimmunoassay, two-dimensional gel electrophoresis (2DE), 2DE followed by mass spectrometry (MS), reverse-phase liquid chromatography (LC), LC-tandem MS, matrix-assisted laser desorption/ionization time-of-flight mass spectrometry (MALDI-TOF MS), and 2DE followed by MALDI-TOF MS [29]. More than 100 salivary molecules have been identified as biomarkers for oral cancer, with the cytokines being the most promising [37]. It was shown that antibodies specific for interleukin (IL)-6 and tumor protein P53 may be potential salivary biomarkers and that IL-6, IL-8, vascular endothelial growth factor, IL-1β, and tumor necrosis factor-alpha (TNF-α) also participate in the initial process of OSCC [38]. Nagler et al. [39] reported a fourfold increase in three known salivary markers in patients with OSCC, namely, cancer antigen 125 (CA125), cytokeratin 19 fragment (Cyfra 21-1), and tissue polypeptide antigen despite without statistical significance. The combination of these circulatory epithelial tumor markers with the salivary concentration of carcinoembryonic antigen enhanced the clinical prediction of OSCC development.

Metabolomics is the study of low-molecular-weight compounds in biological systems. Such analysis of the human metabolome, which is composed of multiple metabolites, contributes to the diagnosis and prognosis of OSCC [40]. Various biomarkers (including carbohydrates, enzymes, and metabolites, and the molecules in liquid biopsies) have been assessed using liquid chromatography, nuclear magnetic resonance, enzyme analysis, and MS [41]. HMDB contains 114222 metabolite entries, including water- and fat-soluble metabolites as well as metabolites considered abundant (> 1 μm) or relatively rare (< 1 nm). In addition, 5702 protein sequences were linked to these metabolite entries. HMDB can be used in metabolomics, clinical chemistry, biomarker discovery, and other studies [42]. Putrescine, which is a cadaverine-related polyamine produced by the breakdown of amino acids, has been found to be at a significantly higher level in patients with oral leukoplakia and OSCC than in control individuals. The level of putrescine is related to the regulation of tumor growth, and there is a significant difference in its levels between experimental individuals and control individuals [40]. Wang et al. [43] identified 14 salivary metabolites as possible biomarkers, of which eight were upregulated and six were downregulated in patients with OSCC compared with their levels in the healthy control group. Five of the salivary biomarkers (propionylcholine, *N-*acetyl-l-phenylalanine, sphinganine, phytosphingosine, and *S*-carboxymethyl-l-cysteine) in combination yielded satisfactory accuracy (*AUC* = 0.997), sensitivity (100%), and specificity (96.7%) in distinguishing patients at stage I–II OSCC from the control group.

The oral microbiota is one of the most diverse of the microbiotas found in nature [44]. A number of studies have demonstrated that oral bacteria may also play an important role in OSCC, based on observed changes in their abundance in the oral cavity of afflicted patients and their possible involvement in various mechanisms of cancer development (e.g., acceleration of cell proliferation, inhibition of cell apoptosis, and improvement of tumor invasion and metastasis) [45, 46]. In human microbiome studies, 16S rRNA-based next-generation sequencing technology is used to determine the structure and function of culturable and unculturable bacterial communities in different parts of the human body under healthy and diseased conditions. The 16S rRNA gene, which is 1,500 bp in length, is used for the identification of bacterial communities. This gene contains nine hypervariable regions (V1–V9). A single hypervariable region cannot be distinguished among all bacteria. Among V1–V9, the V3–V4 regions exhibit the greatest ability to identify bacterial communities, producing a 500-bp-long PCR amplification product that is commonly used in metagenomic studies [47]. Human papillomavirus (HPV) DNA can be detected in a subset of OSCC, and a series of recent reports have provided clear data revealing differences in HPV DNA positivity and oncogene activity [9]. The abundance of lactic acid bacteria and species of the genus *Lactobacillus* in saliva was found to be significantly higher in patients who had undergone chemoradiation or surgery [47]. The abundance of lactic acid bacteria in saliva was also increased in patients at an advanced TNM stage [47].

Liquid biopsy has emerged as a clinically useful tool, with the regulatory approval of several test solutions demonstrating its utility as a futuristic approach for realizing timely and personalized therapeutic decisions [9]. We have provided current information on various novel liquid biopsy biomarkers, such as oral microbiota and metabolites in saliva ctDNAs/CTCs, in terms of their role in OSCC diagnosis, prognosis, and treatment monitoring. However, larger clinical studies/trials on these biomarkers are required to understand these liquid biopsy components in more detail and to validate their translation into high-throughput applicable solutions in the clinical setting. The evidence gathered on these molecules will help advance the use of liquid biopsies for achieving accurate, personalized, and specific guidance in OSCC management.

## Light-based detection system

Light-based detection systems based on the optical properties of biological tissues have emerged as a viable diagnostic option, with reports demonstrating their abilities to improve oral mucosal examination and increase the detection of PMDs and OSCC [48]. The assumption behind optical detection systems, such as chemiluminescence and self-fluorescence imaging, is that tumors and precancerous tissues that undergo abnormal metabolic or structural changes have different absorption and reflection properties when exposed to specific wavelengths of light. Such technologies for oral use have been adapted and marketed over the last decade (chemiluminescence: ViziLite®, ViziLite® Plus, MicroLux DL; spontaneous fluorescence: the visually enhanced light lens VELscope®) [49, 50].

### Chemiluminescence

**Fig. 3:**
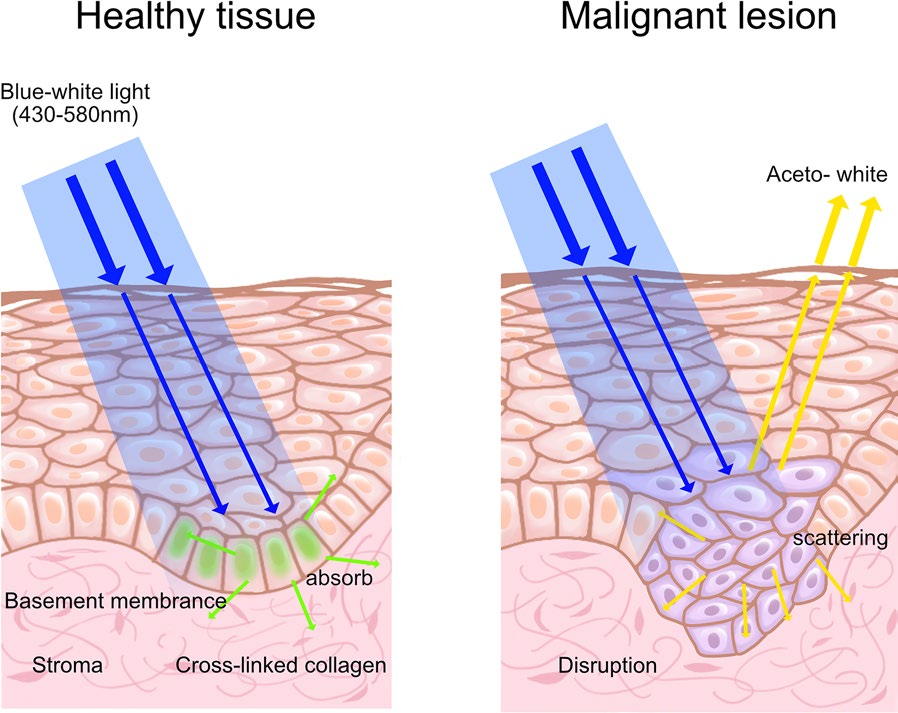
Mechanism underlying the chemiluminescent light system. The color of healthy tissues is dark blue, whereas that of malignant lesions is “aceto-white.” These optical properties result from the different manners in which malignant lesions and normal mucosa scatter light [52]

The term “chemiluminescence” describes the blue/white light (430–580 nm) produced by the chemical reaction of acetylsalicylic acid and hydrogen peroxide inside a capsule light rod. The reaction is based on how light is reflected by tissues that undergo biological changes, including increased nuclear/cytoplasmic ratios (Fig. 3) [51]. The lesion is clear in outline, whereas the normal tissue is darker [49]. A chemiluminescence-based detection tool called ViziLite® (Zila Pharmaceuticals, Phoenix, AZ, USA) is intended to help in the early detection of oral potentially malignant lesions (OPMLs) and OSCC. In a study by Vashisht et al. [52], 60 patients were screened using ViziLite®, that is, 25 patients with potential malignant epithelial lesions (10 of whom specifically had oral leukoplakia), 10 patients with OSCC, and 25 high-risk patients with no clinically visible lesions. Open biopsy was performed. The results revealed that ViziLite® exhibited a diagnostic sensitivity of 95.45% and a specificity of 84.6% and was able to detect early epithelial dysplasia in a high-risk patient with a clinically normal oral mucosa.

In a cross-sectional study, Mehrotra et al*.* [53] compared the ViziLite® Plus and VELscope systems (LED Dental, White Rock, British Columbia, Canada) with conventional oral examination to assess their clinical usefulness in identifying oral lesions and found that neither system demonstrated superiority to the conventional approach. Currently, the main drawback of ViziLite® is the high percentage of false-positive and false-negative test results obtained in the identification of dysplastic areas. ViziLite® facilitates the identification of hyperkeratotic areas and may increase the visibility of mucosal lesions [54].

The new ViziLite® Plus gadget incorporates the diagnostic capabilities of the toluidine blue marking system. The results have been encouraging, demonstrating that toluidine blue could decrease the number of false-positive instances while maintaining the false-negative rate [55]. Although some research studies have concluded that ViziLite® Plus is ineffective for detecting malignancies in patients with clearly visible lesions [56], other lines of evidence support its higher diagnostic accuracy for toluidine blue staining alone [52, 57].

### Tissue autofluorescence

**Fig. 4:**
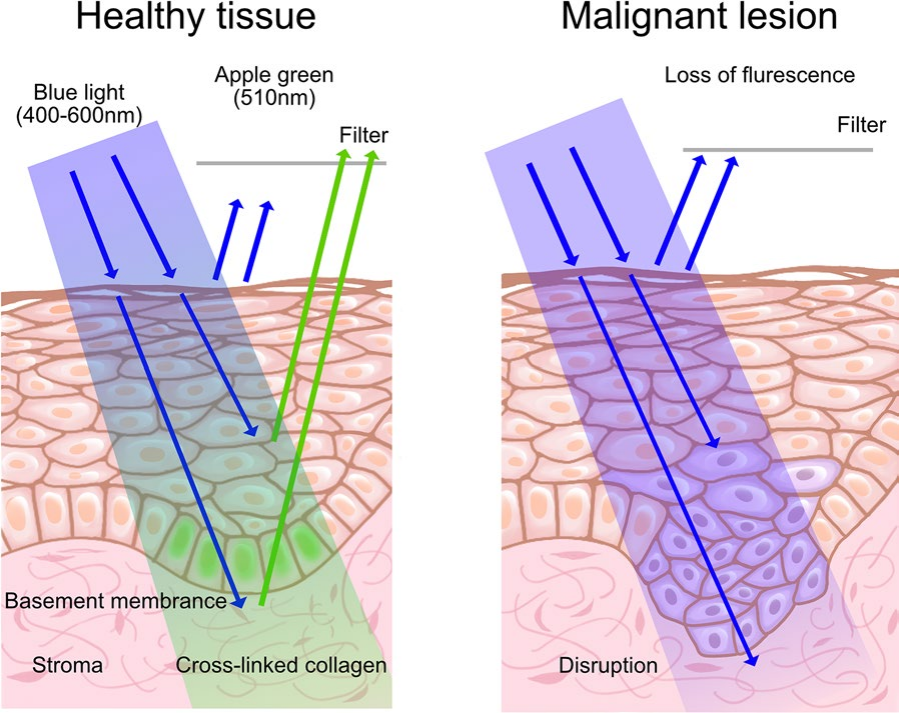
Autofluorescence-based diagnostic technique. Apple green fluorescence (510 nm) is visible in healthy tissues under blue light at 400–460 nm, whereas malignant tumors lose their autofluorescence. This is related to the filtering effect, autofluorescence of collagen cross-links, and redox ratio [52]

Tissue autofluorescence is caused by the stimulation of endogenous fluorophores (e.g., certain amino acids, metabolites, and structural proteins) by an external light source. The most important fluorophores in the oral mucosa are nicotinamide adenine dinucleotide (NADH) and flavin adenine dinucleotide (FAD) in the epithelium and cross-linking collagen in the stroma (Additional file 1: Figure S1). Fluorophores absorb photons from external light sources and emit lower-energy photons, resulting in fluorescence [58]. Owing to the interruption of the distribution of fluorescent pigments, dysplastic tissues lose their fluorescence emission ability and appear darker in color than the surrounding healthy tissues [59] (Fig. 4). VELscope®, which was marketed after FDA approval in 2006, is a handheld nonamplifying device used for the direct observation of oral mucosa self-fluorescence [60]. The lack of a need for technical measures, such as the use of dim lighting, pre-washing, and stain marking solutions, renders VELscope® easy to use, and the emitted light reaches the oral mucosa and stimulates endogenous self-fluorescent fluorophores [61]. Scheer et al. [62] studied 41 patients (19 women and 22 men) with a history of OSCC. After the clinical evaluation, the VELscope® device was used for examination and recording, and then, an incision biopsy was performed. The results revealed that the sensitivity and specificity of VELscope® in detecting oral malignant lesions by autofluorescence were 33.3% and 88.6%, respectively, with a positive predictive value of 33.3% and a negative predictive value of 88.6%. There was no statistical correlation among sex, lesion appearance, and autofluorescence loss. Nevertheless, there is a possibility of overdiagnosis with VELscope® if used by nonspecialists [58]. Moreover, low specificity values were obtained in various studies on individuals with PMDs or OSCC, suggesting possible VELscope® limitations [63–65]. The specificity of VELscope® for PMD and OSCC detection may be increased by combining the system with additional diagnostic procedures. For instance, Kaur et al*.* [66] used salivary protoporphyrin IX levels together with autofluorescence to distinguish between normal mucosa and high-risk lesions.

## Optical coherence tomography

Optical coherence tomography (OCT) is another light-based method for the detection and assessment of OPMLs and OSCC, providing cross-sectional images of biological tissues through optical reflection measurements [67]. The noninvasive nature of this imaging modality combined with (i) a penetration depth of 2–3 mm, (ii) high-resolution (5–15 µm) real-time image viewing, and (iii) the ability to yield cross-sectional and 3D sectional images provides good prerequisites for live oral screening and diagnosis [68]. In OCT scanning systems, low-coherence light is coupled with a fiber-optic Michelson interferometer that effectively captures 2D and 3D images of micron-scale resolution from light-scattered biological tissues [69]. OCT probes can be placed onto the tissue surface to generate surface and subsurface images of the microscopic tissue anatomy and cell structure in real time, thereby avoiding the discomfort, delay, and expense of biopsy [68]. In their study of in vitro tissue samples from 14 patients with OSCC, Yang et al. [70] reported that the diagnostic sensitivity and specificity of OCT detection reached 97.88% and 83.77%, respectively, with a recognition accuracy of 91.15%. Preliminary results suggest that the in-depth mining of hidden information in OCT images can accurately guide the removal of oral cancers, thereby further promoting the clinical application of this diagnostic technique.

Several OCT systems have been approved by the FDA for clinical use, and this diagnostic method is an important imaging modality in ophthalmology. However, despite its superior diagnostic accuracy and enhanced user interface, there are several limitations to its adoption for clinical application, such as the steep learning curve in reading and interpreting the images, unfriendly operating software and user interfaces, and high cost of the system [71].

## Nano detection systems

Nano detection technologies are regarded as novel noninvasive approaches. The manipulation of materials with a length scale of 1–100 nm in at least one dimension is referred to as nanotechnology [72]. Nanoparticles of noble metals, such as gold and silver, exhibit unique enhanced optical properties, owing to the surface plasmon resonance that results from the collective oscillations of resonant electrons on the particle surface with incident light. The size, shape, composition, and surrounding microenvironment are all key factors that determine the enhancement of the photoelectric performance of these metal nanoparticles [73]. By modifying the manufacturing processes, surface chemistry, or performance aspects of nanoparticles, such as their biocompatibility, function-specific size and shape, blood circulation half-life, and targeting of certain cell surface components, they can become effective diagnostic agents [74]. Nanotechnology can be applied to oral cancer detection methods, molecular imaging (magnetic resonance imaging, OCT, photoacoustic imaging, and surface plasmon resonance scattering), and nanoscale ultrasensitive biomarker detection [75]. Legge et al. [76] combined anti-αVβ6-targeted magnetic nanoparticles with thermal ablation and showed the system to be a promising treatment for OSCC. In that study, biocompatible silica-coated magnetic iron oxide nanoparticles were prepared and combined with antibodies targeting αVβ6 integrin, a cell surface biomarker of OSCC. In tissue biopsies from patients with OSCC, the expression of αVβ6 integrin is upregulated compared with its level in normal tissue. Functionalization of the silica coating with the αVβ6 antibodies enabled the nanoparticles to directly target αVβ6-overexpressing cells, resulting in significantly improved killing of the targeted tumor cells by the thermotherapy compared with that of the control group.

Nanoscale agents provide a clearer image, increase the penetration depth, and exhibit enough signals and subcellular spatial resolution. Additionally, the less harmful nano-based contrast agents used for magnetic resonance imaging, OCT, and photoacoustic imaging have a prolonged blood circulation half-life for targeting certain cell surface molecules. Thus, nano-based diagnosis shows promise in providing real-time, convenient, and cost-effective detection services for patients with oral cancer. However, these technologies are still only being tested in animal models or ex vivo studies, and clinical trial information needs to be collected [75].

## Artificial intelligence

The use of artificial intelligence (AI) approaches to enhance image-based diagnosis is becoming more popular [77], with the two main divisions being deep learning and machine learning. Deep learning networks rely on layers of artificial neural networks (ANNs) to generate their own categories, based on their identification of edges (differences) within neural network layers when exposed to a large number of data points. By contrast, machine learning algorithms typically demand an accurately categorized data input. The efficiency of automated cancer diagnosis has significantly increased, owing to the invention and improvement of convolutional neural networks [78]. To put it succinctly, preprocessing, picture segmentation, and postprocessing are the three essential processes for implementing AI in medical imaging. For each site scanned, the clinical and pathologic diagnoses were compared with the AI diagnosis. Compared with the histological analysis, the AI system’s spatially resolved diagnostic accuracy was 92.2%, with 100% sensitivity and specificity for identifying malignancy within the clinically characterized tumor and tumor margin areas [71]. More significantly, AI methods can assess complicated images quickly and offer assistance on decision-making.

## Oral brush cytology

Oral brush cytology (OBC) is a minimally invasive and safe method for extracting cells from the oral mucosa. In addition to identifying potential biomarkers, this technique is useful for screening and early screening purposes [79, 80]. The primary advantage of OBC is that it is a simple, minimally invasive, and relatively painless technique for representative cell diagnosis of the oral mucosa [81]. Data indicate that a cytobrush was the most commonly used tool, followed by the OralCDx® brush. Infant toothbrushes were also used in some areas (India) [80]. Velleuer et al. [82] performed a statistical analysis of 737 lesions, including 86 lesions in 30 patients with at least high-grade oral epithelial dysplasia. The sensitivity and specificity of the OBC method were 97.7% and 84.5%, respectively, whereas those of DNA ploidy analysis were higher at 100% and 92.2%, respectively. Sunny et al. [83] investigated the clinical utility and efficacy of remote cytology systems and ANN-based risk stratification models for the early detection of OPMLs. The overall accuracy of Cellscope in detecting oral lesions was 84–86%, and there was no difference between distal cytology and conventional cytology (kappa value = 0.67–0.72). However, because of the limitations of conventional cytology, its detection sensitivity for OPML is low (18%). The detection sensitivity for malignant lesions (93%) and high-grade OPMLs (73%) was increased via the construction of a combined image processing and ANN-based risk stratification model, leading to an overall accuracy improvement of 30%.

## Other detection methods

Microfluidic systems can act as a miniature automated version of an integrated experimental program on a single device—often referred to as a “lab on a chip” [84]—and can be used for the screening of oral cancer. The term “microfluidic” describes the guided control of fluid flow that is physically limited to small objects [85]. By making analytical systems smaller and more automated, microfluidic systems were enhanced with the goal of acquiring certain properties of traditional analytical procedures. With typical internal capacities ranging from microliters to picoliters, microfluidic instruments are used to handle and modify fluids at a submillimeter scale from 1 to 1000 um [86]. In the early treatment of oral cancer, microfluidic systems are used to determine the risk of mucositis before disease development. In one study, four biomarkers (TNF-α, IL-6, IL-1β, and C-reactive protein) were selected and captured using 1 μm magnetic beads coated with antibody and enzyme labels in the capture chamber [87]. The close correlation between the serum levels of the four biomarker proteins and standard ELISA results for patients with head and neck cancer demonstrated the accuracy and diagnostic utility of this array [87].

Toluidine blue staining has been used in the diagnosis of oral cancer for many years. Staining of the oral mucosa of patients with OPML, using 1% toluidine blue for 30 s, can help distinguish normal tissues from malignant lesions [88]. In a hospital-based study that examined the accuracy of several diagnostic tests, Vijayakumar et al. [89] performed toluidine blue staining on 55 patients with oral mucosal disease, including potential precancerous or malignant lesions, and compared the results with histopathological findings. The sensitivity and specificity of toluidine blue staining were 92.6% and 67.9%, respectively, and its overall diagnostic accuracy was 80%. In another study, toluidine blue staining was used to screen 60 patients, including 25 with OPMLs (10 with oral leukoplakia), 10 with clinically diagnosed OSCC, and 25 high-risk patients without clinical lesions [52]. These patients were then tested with open biopsies. The results revealed the sensitivity and specificity of toluidine blue staining for the diagnosis of this study group to be 86.36% and 76.9%, respectively [52].

Chromosome copy number aneuploidy or abnormality has long been considered to be a determinant of cancer [90]. As a cellular equivalent, DNA can be used for the detection of chromosomal aneuploidy (DNA aneuploidy). Maraki et al. [91] analyzed 98 patients with potential oral lesions using cytology and DNA analysis, and compared the findings with biopsy results. Cytological detection combined with DNA analysis had a diagnostic sensitivity of 100% and specificity of 97.4%, revealing it to be a highly sensitive, specific, and noninvasive method for the early diagnosis of oral epithelial tumors. Moreover, patients showed better compliance with this method.

## Limitations of the above-mentioned methods

In general, these diagnostic methods are minimally invasive, painless, rapid, and economical, and their sensitivity and specificity indicate their excellent application prospects. However, to date, they have all had limited application. For instance, compared with other tumor anatomical sites, liquid biopsy of OPMLs or OSCC in a clinical context is still in its infancy, and future research efforts should focus on sizable, prospective, multicenter trials [9]. Tissue autofluorescence and chemiluminescence techniques have shown limited capacity in recognizing high-risk lesions. For routine screening of OPMLs, conventional visual examination is more reliable than autofluorescence testing using a visually enhanced light scope. The establishment of routine examinations of the entire oral cavity remains the gold standard for the early detection of OPMLs [49]. Although OCT is a noninvasive in situ imaging technique that can obtain images close to histopathological resolution and exhibits great potential for intraoperative diagnosis, it is still impossible to accurately determine the surgical margin solely by direct observation of the qualitative characteristics of the images [92]. Most preoral malignancies are often associated with hyperkeratosis, which has a negative impact on image quality [92]. Additionally, when OCT produces intense light absorption or scattering due to bleeding, it results in intense light attenuation and shadows of deeper structures, thereby reducing the diagnostic value of the final image [93]. With regard to OBC, some studies have demonstrated poor cytological sensitivity and specificity of the brushes, suggesting that this technique should not be used. By contrast, liquid-based brush cytology for the diagnosis of OPMLs and OSCC has yielded promising results. Therefore, clear cytological diagnostic criteria and accurate cytological and histopathological studies are required to confirm the effectiveness of oral liquid-based brush cytology [82]. Further research and exploration are also necessary to verify the use of microfluidic systems for oral cancer detection, which has rarely been reported.

## Conclusions

Herein, we have summarized the various methods used in recent years for the early screening of oral cancer, including liquid biopsy, optical detection systems, OCT, nanotechnology, and OBC (Table 1). These methods test the oral cavity from different dimensions or perspectives and possess the advantages of being minimally invasive, painless, fast, and economical diagnostic techniques with great clinical application prospects. However, each method has its own disadvantages and necessitates further research and breakthrough before these methods can be applied effectively for the early diagnosis of oral cancer.

**Table 1:** Current noninvasive advances in diagnosis of oral squamous cell carcinoma

Test name	Sample	Biomarkers detected	Technology	Application
Liquid biopsy	Blood/Saliva	CTC with EpCAM,CD45 (-) [15]	CellSearch: CTC immuno-isolation and detection by immune-fluorescence	Prognostic treatment assessment
	Plasma	CfDNA/CtDNA	PCR	Prognostic
	Blood/Saliva	Exosomes with miR-34a-5p [23], miR-210-3p [24]	PCR	Prognostic
	Supernatants of Oral mucosal cells	Exosomal metastasis (THBS1) [25]	Flow cytometry, luminex assays; PCR	Treatment
	Oral fluid	CD63, CD9, and CD81 [27]	ELISA and WB	Diagnostic
	Saliva	Hypermethylation of the NID2 and HOXA9 [34]	Genomic analyses	Diagnostic
	Cell	CUL3,ZFP36L2,PTCH1 and ATF5 [35]	WGS and (ChIP)-seq	Diagnostic treatment
	Saliva	A-amylase, cystatin, proline-rich peptide, serum albumin, and mucin [36]	Spectrometry	Diagnostic
	Saliva	IL-6, IL-8, VEGF, IL-1β, and TNF-α [37]	ELISA	Diagnostic
	Saliva	cancer antigen 125 (CA-125), cytokeratin 19 fragment (CyFRA21-1), and tissue polypeptide antigen (TPS) + CTCs [39]	Immunoradiometric assay	Diagnostic
	Saliva	propionylcholine, acetylphenylalanine, sphinganine, phytosphingosine, and S-carboxymethyl-L-cysteine [43]	Spectrometer	Diagnostic
	Saliva	The bacterial 16SrRNA gene contains nine hypervariable regions (V3–V4) [47]	NGS	Diagnostic
LBDS	Oral mucosa	Chemiluminescence	ViziLite®, ViziLite Plus [52–57],	Diagnostic
		Tissue autofluorescence	Fluorescence:VELscope®[58, 62–66]	Diagnostic
OCT	Oral mucosa		In OCT scanning systems, low-coherence light is coupled with a fiber-optic Michelson interferometer that effectively captures 2D and 3D images with micron resolution from light-scattered biological tissues	Diagnostic
Nano detection systems	Oral mucosa	Nanotechnology-based molecular imaging (such as magnetic resonance imaging [MRI], optical coherence tomography [OCT], photoacoustic imaging, and surface plasmon resonance scattering) [75]		Diagnostic
		Nanotechnology-based biomarker αVβ6	Magnetic iron oxide nanoparticles, with a biocompatible silica coating, were produced and conjugated with antibodies to target integrin αvβ6 [76]	Treatment
Oral brush cytology	Oral mucosa		OralCDx®brush[80]	Diagnostic
Microfluidic systems	Saliva		Microfluidic tools handle and manipulate fluids at a submillimeter scale from 1 to 1000 μm, with typical internal volumes of microliters to picoliters [86]	Diagnostic
		Microfluidic systems-based biomarkers TNF-α, IL-6, IL-1β, and CRP [87]	ELISA	Diagnostic

## Supplementary Information

**Additional file1: Fig. S1.** Cell metabolism. This phenomenon is related to the Warburg effect. Malignant lesions dominate reductive glycolysis, leading to the decrease in the redox ratio. TCA: tricarboxylic acid cycle [49].

## Data Availability

Not applicable.
