# Supplementary material for: Current advances in noninvasive methods for the diagnosis of oral squamous cell carcinoma: a review
Source: Eur J Med Res. 2023 Jan 27;28:53. doi: 10.1186/s40001-022-00916-4 (PMC9880940; doi:10.1186/s40001-022-00916-4)
Supplement: Supplementary file 1 — Additional file1: Fig. S1. Cell metabolism. This phenomenon is related to the Warburg effect. Malignant lesions dominate reductive glycolysis, leading to the decrease in the redox ratio. TCA: tricarboxylic acid cycle [49]. [file 40001_2022_916_MOESM1_ESM.docx]

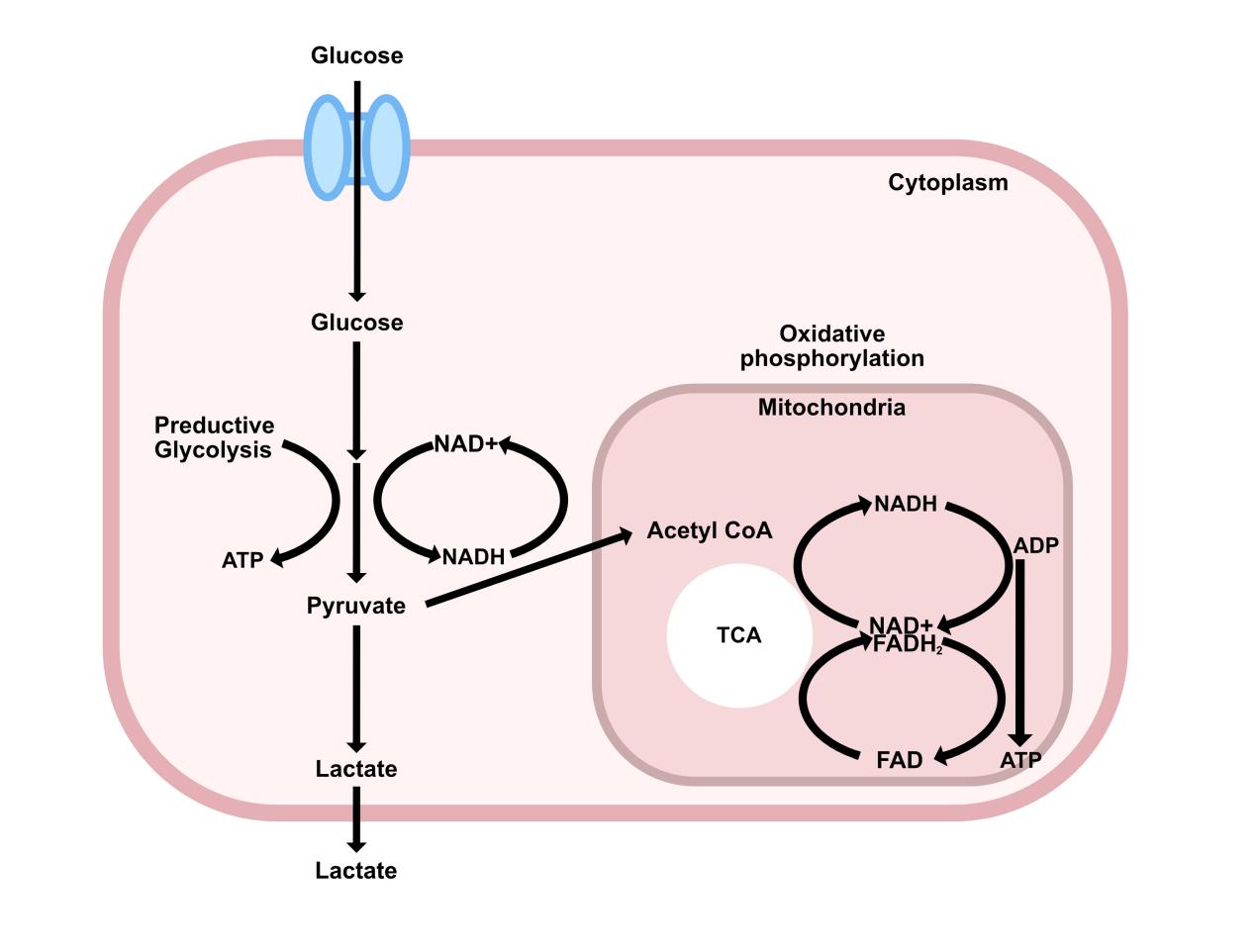


Additional file 1: Fig. S1:Cell metabolism. This phenomenon is related to the Warburg effect. Malignant lesions dominate reductive glycolysis, leading to the decrease in the redox ratio. TCA: tricarboxylic acid cycle [49].
